# Reply to Comment(s) on “Nationwide randomised trial evaluating elective neck dissection for early stage oral cancer (SEND study) with meta-analysis and concurrent real-world cohort”

**DOI:** 10.1038/s41416-020-0983-7

**Published:** 2020-07-16

**Authors:** Iain Hutchison, Allan Hackshaw

**Affiliations:** 1 CE Saving Faces – The Facial Surgery Research Foundation, Consultant Oral and Maxillofacial Surgeon Ipswich Hospital, Hon Consultant Oral and Maxillofacial Surgeon Barts Health NHS Trust, London, UK; 2 Deputy Director, University College London, Cancer Research UK & UCL Cancer Trials Centre, London, UK

**Keywords:** Cancer, Oncology

**Ref to D'Cruz and colleagues**

We thank Dr Anil K. D’Cruz and colleagues^1^ for their positive comments on our paper.^2^

We agree that all head and neck oncologists should now accept the findings of their study and ours, including the meta-analysis, as “irrefutable evidence” in favour of END’s survival benefit in the smallest mouth cancers and should concentrate on more compelling issues for research.^2^ Therefore it is important that we now put effort into achieving this change in guidelines, using evidence on the survival benefit, quality of life and adverse events.

**Ref to Subash and colleagues**

We thank Dr Anand Subash and colleagues^3^ for their kind comments.

We agree that the benefit of END not only lies in removing obvious neck node metastases but also clearing the micro-metastases and isolated tumour cells that are not picked up by standard pathological examination. They point out that these can only be identified using intensive preparatory serial sectioning and immunohistochemistry. We do not have the resources to undertake this work which would probably capture many more patients who only had these microscopic deposits and possibly demonstrate even greater benefit for END.

They comment on the numbers of patients who were treated with subsequent adjuvant chemoradiotherapy.^2^ A major advantage for END is that it also acts as the definitive staging procedure identifying patients with neck metastases who would benefit from adjuvant chemoradiotherapy. This treatment should then be given immediately after surgery at the earliest opportunity and would form part of the patient’s first treatment (within 6 weeks of surgery). In our study similar numbers of patients in each arm received chemoradiotherapy. However more patients in the resection only arm received this treatment when the recurrent disease developed a long time after their surgery.^2^ Therefore, an added advantage of END may lie not only in clearing the metastases but also in using appropriate upfront adjunctive chemoradiotherapy before recurrence. Overall survival is potentially influenced by additional therapies used during follow up and is a feature of most cancer treatment trials. Disease-free survival is an equally good outcome, for which we showed a clear benefit.

Although our clear margin status was indeed lower than the Tata Memorial study our recurrence rates were similar. This reaffirms our conclusion that the SEND trial complements that from the Tata Memorial, a renowned specialist centre for oral cancer, whilst our study was done in 25 hospitals so is generalisable to any setting.^2^ Even with differences in tobacco use between our patient groups END was shown to benefit all patients with T1/T2N0 regardless of aetiology and any potential differences in tumour biology.

## Data Availability

Data and materials will be made available to those requesting it through a formal collaboration.
